# Influences of the Periodicity in Molecular Architecture on the Phase Diagrams and Microphase Transitions of the Janus Double-Brush Copolymer with a Loose Graft

**DOI:** 10.3390/polym14142847

**Published:** 2022-07-13

**Authors:** Dachuan Sun, Yang Song

**Affiliations:** 1 School of Transportation Engineering, Shandong Jianzhu University, Jinan 250101, China; 2 Huzhou Nanxun District Jianda Ecological Environment Innovation Center, Shandong Jianzhu University, Jinan 250101, China

**Keywords:** Janus double-brush copolymer, architectural periodicity, phase diagram, order–disorder transition, self-consistent mean field theory

## Abstract

The backbone of the Janus double-brush copolymer may break during long-term service, but whether this breakage affects the self-assembled phase state and microphase transitions of the material is still unknown. For the Janus double-brush copolymers with a periodicity in molecular architecture ranging from 1 to 10, the influences of the architectural periodicity on their phase diagrams and order–disorder transitions (ODT) were investigated by the self-consistent mean field theory (SCFT). In total, nine microphases with long-range order were found. By comparing the phase diagrams between copolymers of different periodicity, a decrease in periodicity or breakage along the copolymer backbone had nearly no influence on the phase diagrams unless the periodicity was too short to be smaller than 3. For copolymers with neutral backbones, a decrease in periodicity or breakage along the copolymer backbone reduced the critical segregation strengths of the whole copolymer at ODT. The equations for the critical segregation strengths at ODT, the architectural periodicity, and the volume fraction of the backbone were established for the Janus double-brush copolymers. The theoretical calculations were consistent with the previous theoretical, experimental, and simulation results.

## 1. Introduction

Bottlebrush copolymers have various molecular architectures [1,2,3,4,5], such as Janus, block, random, core-shell, and brush-on-brush, etc. Among them, the Janus double-brush copolymer has many pairs of double-brush chains along its backbone, which is one of the most suitable architectures to study the effect of architectural periodicity on the phase behaviors of the copolymer. The Janus double-brush copolymers could be synthesized via graft-from, graft-to, and graft-through methods [6,7,8]. They can self-assemble into various ordered morphologies, such as cylinder and lamellae [7,9], which could be used as templates for micro-electronics and nanomaterials [10]. Moreover, the Janus double-brush copolymers could be used as giant surfactants [11] to stabilize biphasic emulsion droplets, as they consist of two kinds of graft chain along the backbone. The resulting mini-emulsions exhibited remarkably enhanced stability and higher emulsifying efficiency at much lower concentrations than commonly used diblock or triblock surfactants [11,12]. They could also be used as lubrication, elastomer, antifouling, and templating materials [10,13]. However, the influence of architectural periodicity on the phase behaviors of the Janus double-brush copolymer is still unknown.

The Janus double-brush polymers inevitably undergo backbone fracture under the effect of heat, machinery, ultrasound, and ultraviolet (UV), etc., during long-term service [14,15]. Such backbone scission leads to a decrease in backbone length, molecular weight, and architectural periodicity [14,15]. However, how this reduction affects the self-assembled microphases and the order–disorder transitions (ODT) has not been fully investigated. Molecular chain scission and phase changes are helpful when used in environmental stimulus-triggered drug delivery. Variances in phase behaviors with architectural periodicity have been found in many copolymers [16,17]. For example, Tulsi and Simmons [17] investigated the single-molecule self-assembly behavior of (AB)*_n_* multiblock copolymer. When the architectural periodicity *n* increased from 10 to 100, the aggregate morphology changed apparently from spherical micelles to column, sheet, and necklace types, according to the symmetric ratios of AB. However, when *n* increased from 100 to 200, the aggregate morphology remained basically unchanged. Zhou et al. [16] investigated the A_2*n*+1_B*_n_*C*_n_* bottlebrush copolymers and found that the value of segregation strengths χN should be increased to promote phase separation with the increase in architectural periodicity *n*.

The Janus double-brush copolymer with *n* pairs of grafted chains can be deemed as *n* dicopolymer subunits sequentially linked together by their backbones [18]. The influences of such architectural periodicity on the physical properties of the (AC_2_B)*_n_*copolymers is an interesting question. In this paper, Janus double-brush polymers with architectural periodicity ranging from 1 to 10 were studied. By comparing the phase diagrams and calculating the ODT values under different architectural periodicity, the effects of architectural periodicity on the self-assembly and phase behaviors were studied. The effects of backbone scission during polymer service on the phase transitions of the Janus double-brush copolymers were evaluated. During the long-term service of copolymers, the chemical bonds may be broken along the backbone [14,15,19,20,21], thereby reducing the architectural periodicity and causing changes in the physical properties. Therefore, it is necessary to investigate the effect of the bond connection along the copolymer backbone on their phase diagrams, phase transitions, and other phase behaviors. Such investigation is helpful to the design and synthesis of the desired microstructures self-assembled from the copolymers and helpful to bridge the gap between fundamental molecular understanding and practical materials processing.

The rest of the paper is organized as follows: the model of the Janus double-brush copolymers (AC_2_B)*_n_*and their calculation details are given in the Section 2. Results and discussions related to the microphases, triangle phase diagrams, and the ODT values are shown in Section 3. The conclusions and perspectives are in Section 4. The abbreviations and variables used in this paper are listed in Table A1 and Table A2, respectively, in Appendix A.

## 2. Model and Methods

### 2.1. Model

We referred to the experimentally synthesized (PU-*g*-PDMA/PMMA) [18] to model the Janus double-brush copolymer with similar molecular structures. The (PU-*g*-PDMA/PMMA) copolymer consisted of a polyurethane (PU) backbone and poly(*N,N*-dimethyl acrylamide) (PDMA) and poly(methyl methacrylate) (PMMA) chains grafted simultaneously at the same reactive site along the backbone which were synthesized through the combination of polyaddition and the reversible-deactivation radical polymerization reactions [18]. As the number of repeat units of PU was around 8, referring to its molecular weight (∼10,000 g/mol) measured by gel permeation chromatography (GPC), here we set the architectural periodicity to range from 1 to 10. In this paper, we focus on the case of bond breakage from the backbone. Both the PDMA and PMMA chains are linked by carbon–carbon bonds, while the PU backbone is linked by ester bonds and amide bonds. The PU backbone is more prone to hydrolysis, alcoholysis, and other degradation in humid and alkaline environments, resulting in the fracture of the backbone and a reduction in architectural periodicity. Therefore, the study in this paper and the study of the interfacial properties of the copolymer [18] are complementary to each other.

**Figure 1:**
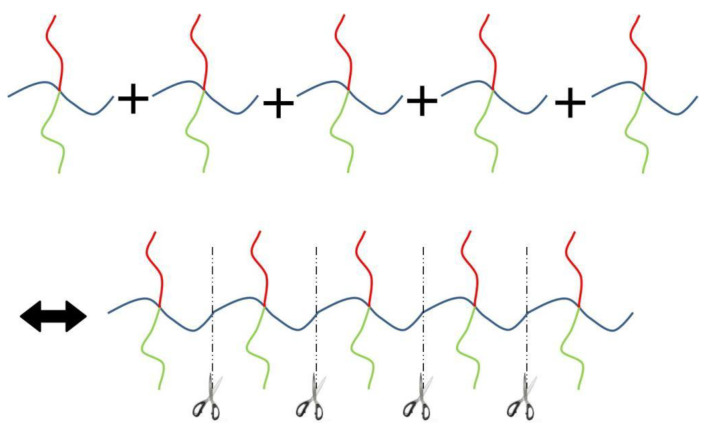
The Janus double-brush copolymer (AC_2_B)*_n_* contains *n* constituting subunits, and each subunit is a four-arm star copolymer. Here the architectural periodicity *n* is 5. Each AC_2_B subunit consists of one arm A chain, one arm B chain, and two arm C chains. In this plot and in the following snapshots, the segments A, B, and C are colored in red, green, and blue, respectively.

Here we consider an incompressible melt of the Janus double-brush copolymers (AC_2_B)*_n_* with chain length *N* and, in total, *m* such chains in a system volume *V*. The topologies of the copolymers investigated by the self-consistent mean field theory (SCFT) are shown in Figure 1; they have *n* constituting block copolymers, and the fundamental unit of architecture is a four-arm AC_2_B star copolymer. The segments A, B, and C are colored in red, green, and blue, respectively. All segments (A, B, and C) have the same segment length and identical diameter σ to simplify the model. The copolymer in Figure 1 can be deemed as *n* = 5, with the four-arm AC_2_B star subunits linked together through their C arms. For each four-arm AC_2_B star subunit, it has two arm C chains each with length *L*_C_, one arm A chain of length *L*_A_, and one arm B chain of length *L*_B_. The compositions of the A, B, and C segments in the copolymers (AC_2_B)*_n_* are denoted as *f*_A_, *f*_B_, and *f*_C_, respectively. In the following, the two arm C chains are supposed to have the same length *L*_C_.

As the ratios of the degrees of polymerization of the PDMA and PMMA graft chains were 48:78, 48:50, and 48:27, respectively [18], in the range of 0.6 to 1.8, in this paper the ratio of the graft chains was changed to a wider range of 1:4 to 4:1 when studying the phase diagrams. Among the three ratios studied, when the ratio of the degree of polymerization of the two graft chains of PDMA and PMMA was 48:50 (around 1:1), the interfacial tension was the lowest; the size of the emulsion was the smallest; and the stability of the emulsion was the best [18]. Therefore, the ratio of the graft chains was set at 1:1 (i.e., $L_{A}=L_{B}$) when studying the ODT of the copolymer in this paper.

In the following, Janus double-brush copolymers with architectural periodicity *n* ranging from 1 to 10 were investigated. During service, the backbone, composed of 1,4-butanediol (BDO) and diphenyl methane diisocyanate (MDI), may break bonds, resulting in a reduction in the number of repeat units on the backbone from 8 to 1, gradually. Therefore, the number of repeat units *n* in the range of 1 to 10 was studied. The unfavorable interactions between segments A, B, and C were labeled by the Flory–Huggins dimensionless exchange energy χ. Two sets of segregation strengths were considered in the following. In the first set, the segments (A, B, and C) were incompatible with each other, and they had the same repulsive strengths between each other $\chi_{\mathrm{AB}}N=\chi_{\mathrm{BC}}N=\chi_{\mathrm{AC}}N=\chi N$. Since the copolymer had three kinds of blocks, the phase space was very large. To simplify the study, we fixed the segregation strengths or their ratios as constant values. $\chi_{\mathrm{AB}}N=\chi_{\mathrm{BC}}N=\chi_{\mathrm{AC}}N=\chi N$ was one of the simplest cases. Here $\chi_{ij}$ was the Flory–Huggins interaction parameter between dissimilar segments *i* and *j*. Compositions *f*_A_, *f*_B_, and *f*_C_ were changed to plot triangle phase diagrams.

In the second set, segregation strengths were set to be $\chi_{\mathrm{AB}}N=\chi N$ and $\chi_{\mathrm{BC}}N=\chi_{\mathrm{AC}}N=0$. Thus, only the A and B segments were incompatible, while the C segments were neutral to the A and B segments. In this case, there was only one ${\left(\chi N\right)}_{\mathrm{ODT}}$, which greatly simplified our research. The entire polymer chain seemed like A-*b*-B diblock copolymers linked together through their junction points by the neutral C arms. Moreover, the lengths of the arm A and arm B chains were set to be equal, $L_{A}=L_{B}$. Thus, it had *f*_A_ = *f*_B_ = (1 − *f*_C_)/2, and the whole chain length *N* could be expressed as $N=n\left(L_{A}+L_{B}+2L_{C}\right)=2n\left(L_{A}+L_{C}\right)$. The relations between ${\left(\chi N\right)}_{\mathrm{ODT}}$ and periodicity *n*, arm C length $L_{C}$, and composition *f*_C_ were investigated.

### 2.2. Calculation Methods

Calculations of the end-segment distribution function *q* and its conjugate $q^{+}$ were the key step in SCFT. The distribution functions satisfied the modified diffusion equation for the Gaussian chains subjected to hypothetical external potentials $\omega_{A}$, $\omega_{B}$, or $\omega_{C}$, which transferred the effect of χ on chain conformations to the partition functions. The conventional SCFT method [22,23,24] was employed to derive *q* and $q^{+}$. The free-energy density F for the incompressible copolymer melt was given as:

(1) $$\begin{array}{ll} \frac{F}{mk_{B}T}=-\ln Q+ & \frac{1}{V}\int dr\left\{N\chi_{\mathrm{AB}}\phi_{A}\left(r\right)\phi_{B}\left(r\right)+N\chi_{\mathrm{AC}}\phi_{A}\left(r\right)\phi_{C}\left(r\right)+N\chi_{\mathrm{BC}}\phi_{B}\left(r\right)\phi_{C}\left(r\right)\right\}\\ & +\frac{1}{V}\int dr\left\{-\omega_{A}\left(r\right)\phi_{A}\left(r\right)-\omega_{B}\left(r\right)\phi_{B}\left(r\right)-\omega_{C}\left(r\right)\phi_{C}\left(r\right)-\eta \left(r\right)\left[1-\phi_{A}\left(r\right)-\phi_{B}\left(r\right)-\phi_{C}\left(r\right)\right]\right\} \end{array}$$

where $k_{B}T$ had its usual meaning of thermal energy. The partition function Q was for a single polymer chain interacting with the mean fields ($\omega_{A}$, $\omega_{B}$, and $\omega_{C}$) produced by the surrounding chains. Moreover, $\phi_{A}\left(r\right)$, $\phi_{B}\left(r\right)$, and $\phi_{C}\left(r\right)$ denoted the local volume fractions at position r for the A, B, and C segments, respectively. The symbol *η* implied the Lagrange multiplier to ensure an incompressibility constraint.

The phase behaviors of comb-like copolymers were analyzed in two dimensions (2D). Their snapshots could be compared with the experimental images taken by transmission electron microscopy (TEM) [25]. The two-dimensional simulation box had size 10$R_{g}$ along the X and Y axes. The spatial coordinate r was scaled by the copolymer’s radius of gyration, $R_{g}$, where $R_{g}^{2}=\sigma^{2}N/6$. The simulation box with periodic boundaries was discretized into 128 × 128 lattice cells. The chain contour of the comb-like copolymer (AC_2_B)*_n_* was discretized into 64*n* lattice points, with each arm C chain discretized into 32 lattice points, while each graft arm A or arm B chain was discretized into 32 lattice points. Larger lattice points were also tested, but they did not change the F values or phase behaviors. The pseudospectral scheme [26] was used to solve the modified diffusion equation. The Fast Fourier Transform in the West (FFTW) [27] was used to accelerate the calculations. All calculations started from random initial states with random initial fields and volume fractions for the A, B, and C segments. The iteration procedure for finding the self-consistent fields was terminated when the free energy was converged within the tolerance level of 10^−12^. Calculations were repeated five times by using different initial conditions to avoid trapping in a metastable state and to ensure that the phenomena were not accidental.

## 3. Results and Discussion

### 3.1. Triangle Phase Diagrams

In this part, several triangle phase diagrams were made for the Janus double-brush copolymers (AC_2_B)*_n_* with architectural periodicity *n* ranging from 1 to 10 in their sequences. The composition *f_i_* increases closer to the corner *i*, where *i* = A, B, and C in the triangle phase diagrams. The A, B, and C segments are incompatible with each other and have the same repulsive strengths between each other $\chi_{\mathrm{AB}}N=\chi_{\mathrm{BC}}N=\chi_{\mathrm{AC}}N=\chi N$. As shown in Table 1, the microphases obtained from the SCFT calculations are: core-shell hexagonal lattice phase (denoted as CSH), three-color lamellar phase (denoted as LAM3), lamellar phase with beads inside (denoted as LAMBD), lamellar phase with alternating beads (denoted as LAMAB), two interpenetrating tetragonal lattice phase (denoted as TET2), hexagon outside “two-color” hexagonal lattice phase (denoted as HEX3I), octagon-octagon-tetragon phase (denoted as OOT), and three-color hexagonal honeycomb phase (denoted as HEX3). Moreover, DIS is used to represent the disordered phase, and LAM2 is used to represent the “two-color lamellar” phase [28,29]. These phases were also found in previous theoretical and experimental reports [16,25,28,29,30,31]. Wang et al. [29] investigated the ABCD 4-miktoarm star copolymer with similar segregation strengths $\chi_{\mathrm{AB}}N=\chi_{\mathrm{AC}}N=\chi_{\mathrm{AD}}N=\chi_{\mathrm{BC}}N=\chi_{\mathrm{BD}}N=\chi_{\mathrm{CD}}N$ in 2D and found more microphases than the (AC_2_B)*_n_* copolymers used here. Sun et al. [28] investigated the H-shaped (AC)B(CA) copolymers with χN=45~125, and the microphase morphologies were shown in three dimensions (3D).

To help interpret the conformational packing of copolymers in these ordered morphologies, schematic plots of copolymer conformations are given in the last volume of Table 1, using a four-arm AC_2_B star copolymer as an example [16,28,30]. After comparing the local $\phi_{i}\left(r\right)$ values, the regions with the main A, B, and C segments are colored in red, green, and blue, respectively. Gumus et al. [32] investigated the self-assembly of mikto-grafted bottlebrush (C-*g*-A*_x_*/B*_y_*)*_n_* in the low-concentration limit, which could complement our work on the molten copolymers.

**Figure 2:**
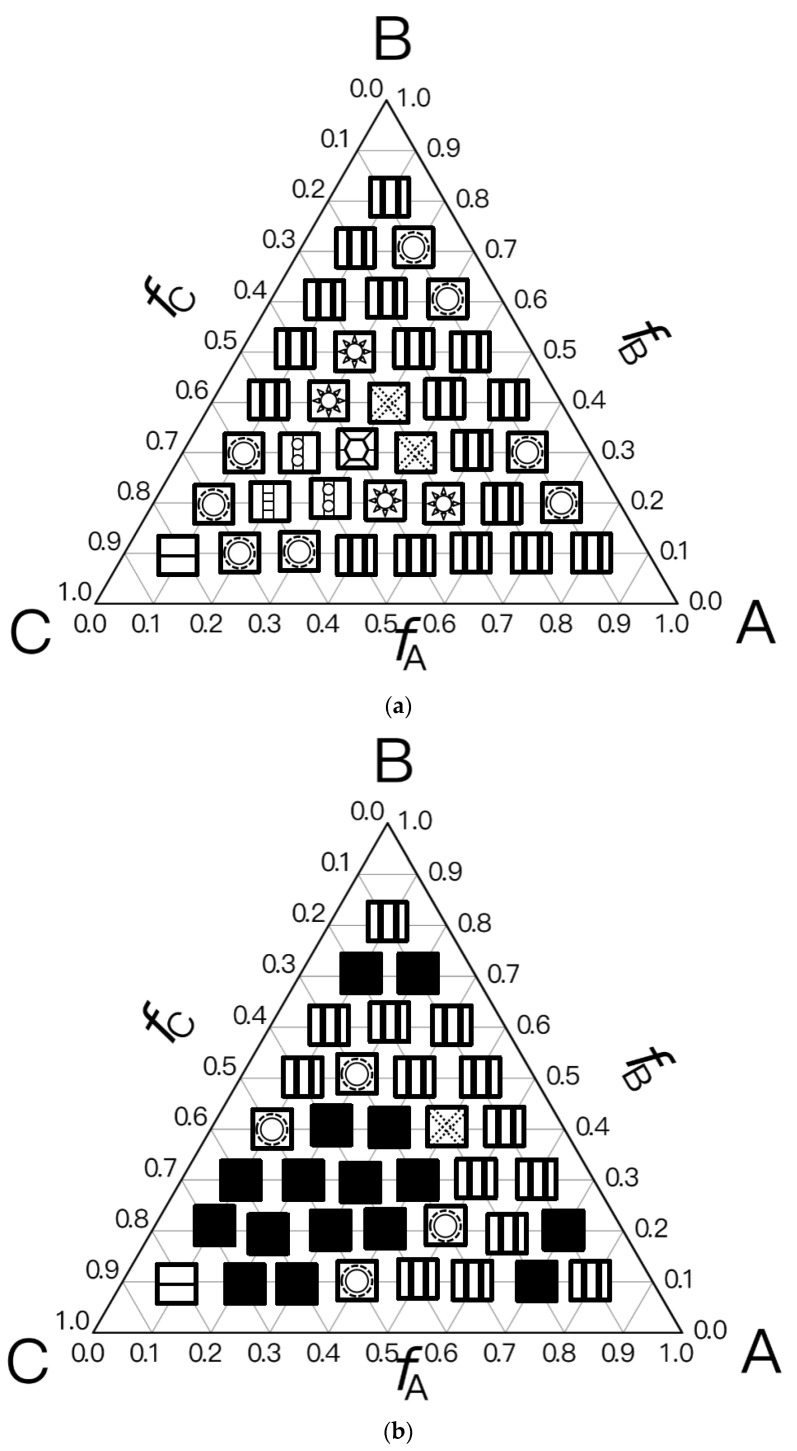

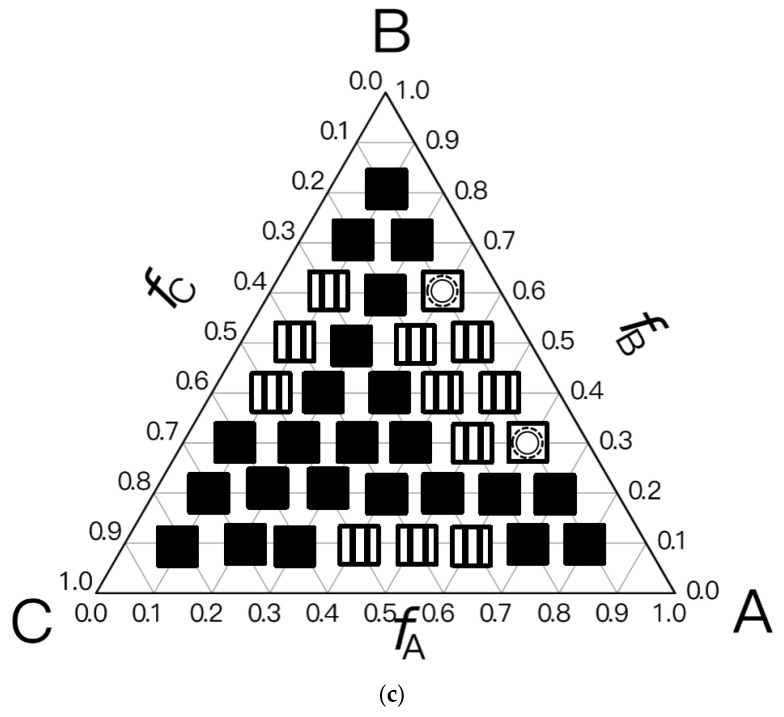
The triangle phase diagrams for the Janus double-brush copolymer (AC_2_B)*_n_* containing *n* subunits in its sequence at different segregation strengths $\chi_{\mathrm{AB}}N=\chi_{\mathrm{BC}}N=\chi_{\mathrm{AC}}N=\chi N$. (**a**) Architectural periodicity *n* = 1 and segregation strength χN=35; (**b**) *n* = 2 and χN=35; (**c**) *n* = 1 and χN=35/2=17.5.

For the simplest case, the Janus double-brush copolymer (AC_2_B)*_n_* has only one subunit *n* = 1, which is a four-arm star copolymer AC_2_B. Its phase diagram at χN=35 is shown in Figure 2a. In total, eight microphases were found for this copolymer. Near the center is the HEX3 phase, in which the segments A, B, and C form similar (hexagonal) phase domains. At the corners of the phase diagram, there are the CSH, LAM2, and LAM3 microphases. Because the segregation strengths are the same for the three pairs, the phase diagram is symmetric when viewed from corner A (large *f*_A_) or corner B (large *f*_B_). The microphase with the largest phase region on the diagram is LAM3, which occupies nearly 50% of the area of the phase diagram, probably owing to the symmetric composition *f*_A_ = *f*_B_. With increasing *f*_C_ at *f*_A_ = *f*_B_, the copolymer transits from the LAM3 microphase to the TET2, HEX3, and LAMBD microphases, then into the LAMAB and CSH microphases, and, finally, the LAM2 microphase occurs.

Experiments indicated that a new microphase would be formed even at the same χN when the copolymer sequence was varied [33]. Our calculation results confirmed this. At the same segregation strength χN=35, Figure 2b was obtained for the Janus double-brush copolymers (AC_2_B)*_n_* with *n* = 2; it is dramatically different from Figure 2a for the Janus copolymers with *n* = 1. One major difference is that nearly 44.4% of the phase region on the phase diagram is covered by the DIS phase. Zhou et al. investigated the ordered microstructures self-assembled from the A_2*n*+1_B*_n_*C*_n_* bottlebrush copolymers [16]. They found that the disordered region was enlarged when the periodic number *n* in the copolymer sequence was increased from 1 to 2 while the χN value was maintained. This is consistent with our calculation results in Figure 2. Moreover, the phase region of the LAM3 microphase drops to 33.3% in Figure 2b. With increasing *f*_C_ at *f*_A_ = *f*_B_, the phase transits from the LAM3 microphase into the TET2 microphase and then becomes disordered before the LAM2 phase finally occurs at the C corner *f*_C_ = 0.8.

In Figure 2c, a phase diagram for the Janus double-brush copolymer (AC_2_B)*_n_* with architectural periodicity *n* = 1 and segregation strength χN=17.5 is shown. Compared to Figure 2b, the copolymers in Figure 2c have the same value of χ, as they have half the total chain length *N* and also the half the segregation strength χN. The major difference is that there is a linkage between the two AC_2_B star subunits in Figure 2b, while such a linkage disappears in Figure 2c. This topological connection causes apparent differences for the two phase diagrams in Figure 2b,c. Similar to Figure 2b, a high coverage of the DIS phase is found on the phase diagram in Figure 2c. However, the areal percentage for the DIS phase increases to 63.8% in Figure 2c. Different from Figure 2b, there are only two microphases (LAM3 and CSH), and the TET2 phase disappears in Figure 2c. The linkage between two star subunits seems to be a prerequisite for the formation of the TET2 phase besides a lower segregation strength. Thus, the linkage between two star subunits could alter the phase behavior of copolymers and change the phase regions of the diagram. Investigation from Wang et al. of the bottlebrush copolymers A*_n_*_+1_(BC)*_n_* reached a similar conclusion [30]. Sun et al. also found that the DIS phase disappeared gradually in the triangle phase diagrams when χN increased from 45 to 90 [28].

**Figure 3:**
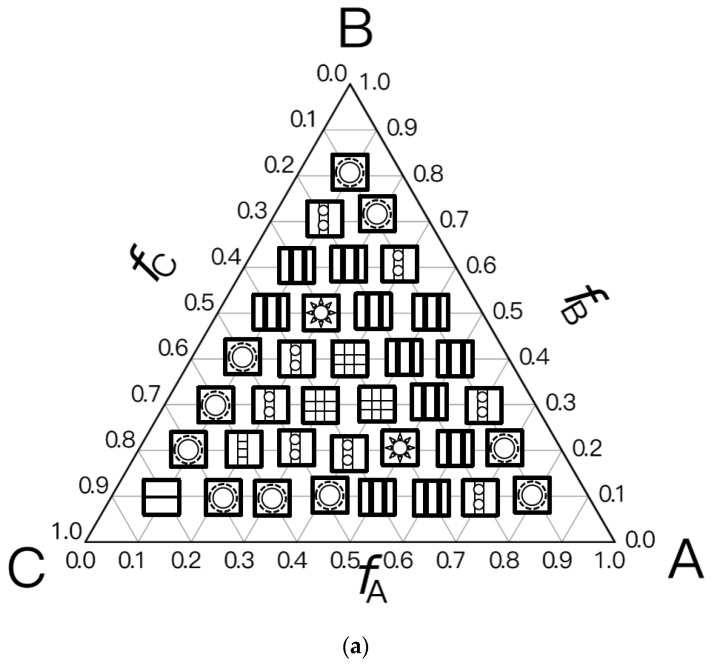

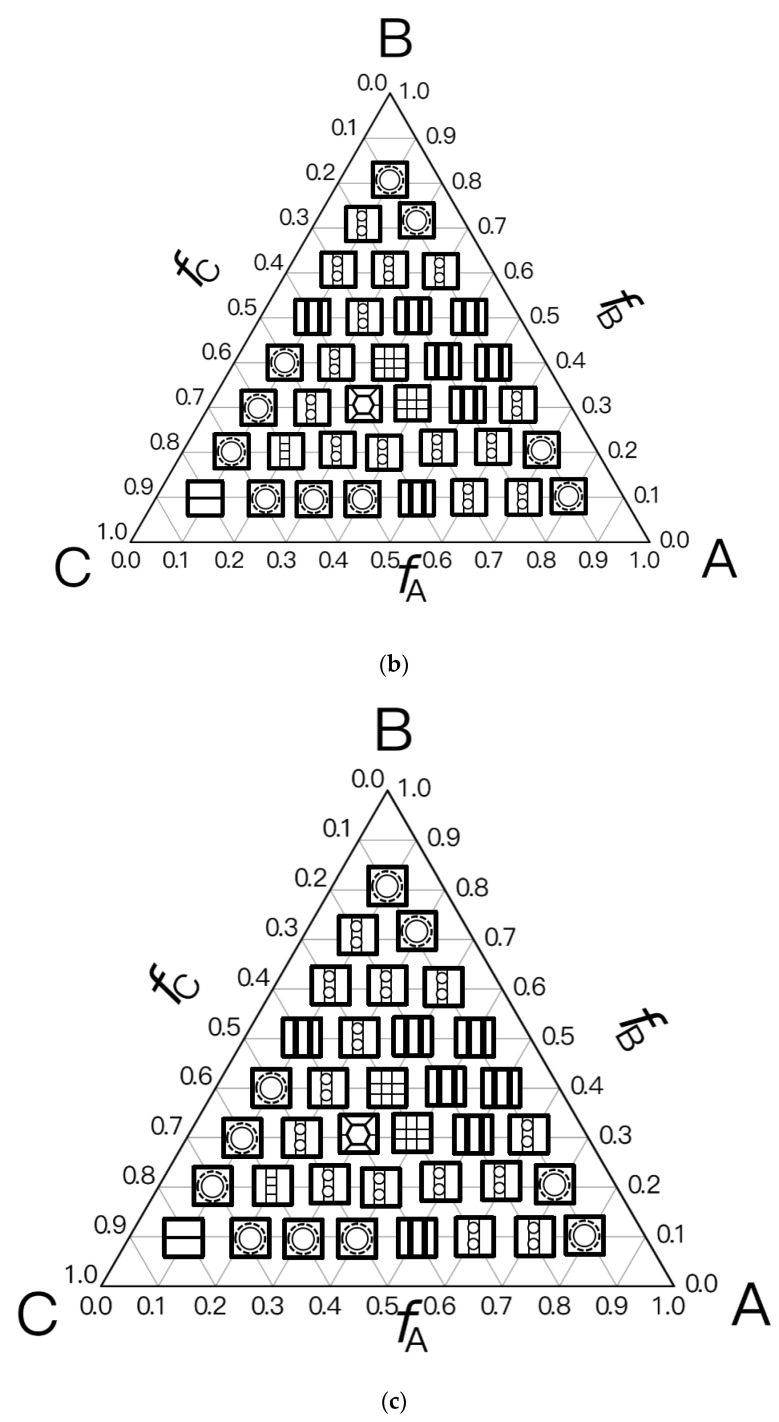
The triangle phase diagrams for the Janus double-brush copolymer (AC_2_B)*_n_* containing *n* star subunits in its sequence at $\chi_{\mathrm{AB}}N=\chi_{\mathrm{BC}}N=\chi_{\mathrm{AC}}N=\chi N$. The χN/n value is maintained at 35. (**a**) Architectural periodicity *n* = 2 and segregation strength χN=35×2=70; (**b**) *n* = 3 and χN=35×3=105; (**c**) *n* = 10 and χN=35×10=350.

For the Janus double-brush copolymer (AC_2_B)*_n_* with *n* = 2, χ could be maintained at the same value when χN was doubled compared to the copolymer (AC_2_B)*_n_* with *n* = 1. Whether these two copolymers had the same phase diagram at the same χN/n value was unknown. To obtain an answer, a phase diagram was made in Figure 3a for the Janus double-brush copolymer (AC_2_B)*_n_* with architectural periodicity *n* = 2 at χN=35×2=70, and it was compared with the phase diagram in Figure 2a for copolymer with *n* = 1 at χN = 35. Several different microphases can be observed through comparison between the phase diagrams, although nearly 66% of the two diagrams is the same. For example, at *f*_A_ = 0.3, *f*_B_ = 0.4, and *f*_C_ = 0.3, the comb-like copolymer with *n* = 2 is the OOT microphase, while the TET2 microphase occurs for the copolymer with *n* = 1. The HEX3I microphase in Figure 2a at *f*_A_ = 0.2, *f*_B_ = 0.4, and *f*_C_ = 0.4 is replaced by the LAMBD phase in Figure 3a. Comparison between Figure 2a and Figure 3a indicates that the phase region for the CSH microphase rises from 22.2% to 27.7%, and the phase region for the LAMBD microphase increases from 5% to 22.2%. The phase region for the LAM3 microphase drops from 41.6% to 30.5%. Therefore, even at the same χN/n value, the topological linkage causes apparent differences in phase diagrams by comparison between the Janus double-brush copolymers (AC_2_B)*_n_* with *n* = 1 and with *n* = 2.

In Figure 3b, the phase diagram for the Janus double-brush copolymers (AC_2_B)*_n_* with architectural periodicity *n* = 3 at tripled segregation strengths χN=35×3=105 is shown. The similarity of the two phase diagrams in Figure 3a,b increases to 86%. At *f*_A_ = 0.3, *f*_B_ = 0.3, and *f*_C_ = 0.4, the OOT microphase changes into the HEX3 microphase. Moreover, several LAM3 microphases transit into the LAMBD microphase. Thus, the phase region for the LAMBD microphase increases to 33.3%, while the phase region for the LAM3 microphase drops to 19.5% on the phase diagrams. Therefore, with increasing *n*, the similarity between neighbor phase diagrams increased, while the influence of topological connection on the phase diagrams decreased for the Janus double-brush copolymers (AC_2_B)*_n_*.

The segregation strengths χN for the Janus double-brush copolymer (AC_2_B)*_n_* were set as χN/n=35 in Figure 3. With further increasing *n* at *n* ≥ 3, however, no apparent changes were found for the phase diagrams. For example, the Janus double-brush copolymer (AC_2_B)*_n_* with architectural periodicity *n* = 5 at χN=35×5=175 has the same phase diagram as (AC_2_B)*_n_* with *n* = 3 at χN=35×3=105. In Figure 3c, the phase diagram for the Janus double-brush copolymer (AC_2_B)*_n_* with architectural periodicity *n* = 10 at χN=35×10=350 is shown; it has no difference with Figure 3b. Therefore, the linkage between the stars AC_2_B improves the critical segregation strength for the occurrence of the microphases and also changes the location of several microphases on the phase diagram. However, such changes gradually fade out with increasing *n*_._ At *n* ≥ 3, *n* has no apparent influence on the locations or phase regions of the self-assembled microphases, according to the phase diagrams for copolymer (AC_2_B)*_n_* at χN=35n. Therefore, the influence of topological connection on the phase diagrams disappears quickly with increasing architectural periodicity *n* for the Janus double-brush copolymer (AC_2_B)*_n_*. In other words, the decrease in periodicity or breakage along the copolymer backbone had nearly no influence on the phase diagrams unless the architectural periodicity was too short to be smaller than 3.

Experimental results from Li et al. indicated that even the single-molecule Janus double-brush copolymer could phase separate after long-time annealing at high temperature [34]. Gao et al. experimentally investigated the Janus double-brush copolymers (AC_2_B)*_n_* as macromolecular emulsifiers with *n* around 8 [18]. Compared with PDMA-*b*-PMMA diblock copolymers, the PU-*g*-PDMA/PMMA Janus double-brush copolymers had a stronger ability to reduce surface tension (from 5.9 to 1.8 mN/m), while they formed a more stable emulsion of a smaller size. Similar to the microphase TET2 in this paper, the PU-*g*-PDMA/PMMA double-brush copolymer adopted “Janus-like” conformations at the interface: the PDMA and PMMA graft chains extended in benzene and [Bmim][PF_6_] phases to avoid the possible unfavorable contact, and PU backbones were entrapped at the interfaces [18]. In the TET2 microphase, the backbones were also at the interface, and the graft chains were distributed in their respective phase regions; thus, the theoretical calculation here is consistent with the experiment. From Figure 2a,b in this paper, the TET2 microphase is formed when the ratio of the two graft chains is equal. Consistent with the theoretical result, the PU-*g*-PDMA_48_/PMMA_50_ copolymer used in the experiment was also equal in the proportion of the graft chains. Asymmetric graft chains resulted in a significant increase in the interfacial tension and a significant increase in the emulsion size with respect to symmetric graft chains [18].

### 3.2. The Critical Values for the Order–Disorder Transitions

The segregation strengths were set as $\chi_{\mathrm{AB}}N=\chi N$ and $\chi_{\mathrm{BC}}N=\chi_{\mathrm{AC}}N=0$ in this part. Arm A and arm B had the same length *L*_A_ = *L*_B_, while the two C arms had the same length *L*_C_. With such symmetric segregation strengths and chain topologies, only the lamellar microphase was found by our calculations. The arm C chains in the Janus double-brush copolymers can be considered as neutral threads linking together several AB diblock copolymers through their midpoints. The total length *N* of the comb-like copolymer now is $N=2n\left(L_{A}+L_{C}\right)$ and *f*_C_ = 1 − 2*f*_A_. To investigate the effect of the arm C length *L*_C_ on ${\left(\chi N\right)}_{\mathrm{ODT}}$, the lengths of arm A and arm B are supposed to be invaried (*L*_A_ = *L*_B_ = *const*), while *L*_C_ is in units of *L*_A_ and denoted as $L_{C}/L_{A}$ in the following. The relationship between the composition *f*_C_ and the ratio $L_{C}/L_{A}$ is, thus, *f*_C_ = (1 + $L_{C}/L_{A}$)^−^^1^.

**Figure 4:**
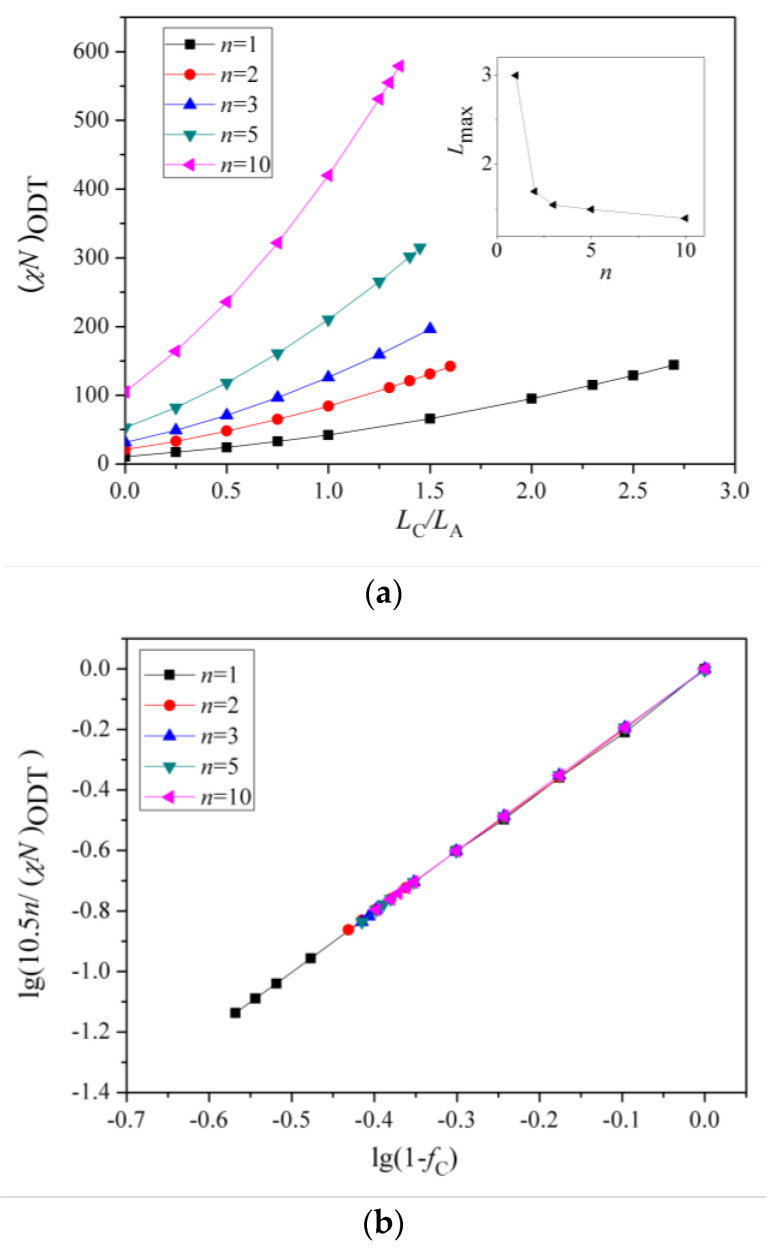
(**a**) The ${\left(\chi N\right)}_{\mathrm{ODT}}$ values for the Janus double-brush copolymer (AC_2_B)*_n_* with different architectural periodicity *n* of star subunits and different ratios between the lengths of the arm C and arm A chains $L_{C}/L_{A}$. (**b**) The double logarithmic plot of the ${\left(\chi N\right)}_{\mathrm{ODT}}$ values versus the composition of the C segments *f*_C_. All data fall on the same line, and the linear line can be fitted nicely by the same formula ${\left(\chi N\right)}_{\mathrm{ODT}}=10.5n{\left(1-f_{C}\right)}^{-2}$.

Above the critical value ${\left(\chi N\right)}_{\mathrm{ODT}}$, the lamellae-to-disorder transitions occur for the symmetric Janus double-brush copolymers (AC_2_B)*_n_*. Results indicate that an ordered microstructure (lamellar phase) cannot be formed when the arm C chains are longer than a critical ratio $L_{C}/L_{A}$ (denoted as $L_{\max}$). When *f*_C_ is low, C segments are distributed between the interfaces of domain A and domain B to shield the repulsive forces between the A and B segments. When the C content *f*_C_ is too high, it may be that too many C segments cause the lamellar structure to be less stable. Moreover, *L*_max_ decreases with *n*, as shown in the inset of Figure 4a. The number of topological connections between the AC_2_B star subunits increases with *n* in the Janus double-brush copolymer (AC_2_B)*_n_*. Moreover, such connections make the arm C chains more inclined to distribute near the interfaces between domain A and domain B, which weakens the repulsion between the A and B segments and makes it more difficult to form an ordered microstructure. Therefore, *L*_max_ decreases with *n*.

When $L_{C}/L_{A}$ is smaller than *L*_max_, the ${\left(\chi N\right)}_{\mathrm{ODT}}$ values for the Janus double-brush copolymers (AC_2_B)*_n_* with *n* = 1, 2, 3, 5, 10 are shown in Figure 4a. The ${\left(\chi N\right)}_{\mathrm{ODT}}$ values increase with *L*_C_ in Figure 4a. As the arm C chain is neutral to both the arm A and arm B chains, increasing the content of arm C chain would reduce the repulsion between the arm A and arm B chains, thus requiring a higher segregation strength for the microphase separation to occur. With increasing *n*, the total chain length *N* increases, as $N=2n\left(L_{A}+L_{C}\right)$. Supposing that the χ value is maintained, a higher χN is required for the microphase separation to occur, because *N* increases with *n*. Thus, the ${\left(\chi N\right)}_{\mathrm{ODT}}$ values increase with both *n* and *L*_C_. Results from the SCFT calculations also indicate that the ${\left(\chi N\right)}_{\mathrm{ODT}}$ values increase with architectural periodicity *n* for the A*_n_*_+1_(BC)*_n_* copolymers [35]. Thus, our results are consistent with the previous theoretical and simulation results.

Figure 4b is the double logarithmic plot of (10.5*n*/${\left(\chi N\right)}_{\mathrm{ODT}}$) versus (1 − *f*_C_). All data fall on the same line, and the slope of the line is 2.0. Thus, the ${\left(\chi N\right)}_{\mathrm{ODT}}$ values for the Janus double-brush copolymers (AC_2_B)*_n_* with different *n* and *L*_C_ values can be fitted by:

(2) $${\left(\chi N\right)}_{\mathrm{ODT}}=10.5n{\left(1-f_{C}\right)}^{-2}.$$

At *f*_C_ = 0, the (AC_2_B)*_n_* copolymers become (AB)*_n_* star polymers. Sanchez et al. [36] found that the star copolymers with equal numbers (*n*) of A and B arms had a critical point at ${\left(\chi N_{0}\right)}_{\mathrm{ODT}}=10.5$ for all values of *n,* with $N_{0}=N/n$. According to Equation (2), ${\left(\chi N/n\right)}_{\mathrm{ODT}}=10.5$ at *f*_C_ = 0, consistent with the previous theoretical results [36]. Wang et al. studied the critical value ${\left(\chi N\right)}_{\mathrm{ODT}}$ of different architectural periodicity values *n* of the A*_n_*_+1_B*_n_* copolymer. With the increase in *m*, ${\left(\chi N\right)}_{\mathrm{ODT}}$ increased, but ${\left(\chi N\right)}_{\mathrm{ODT}}/\left(m+1\right)$ remained constant and was related to the fraction of the A blocks [37]. Palyulin and Potemkin [38] investigated the ODT for melts of a similar Janus double-comb copolymer (AC_1_B)*_n_*. The backbone units served as a nonselective solvent, and the Flory—Huggins parameters in the spinodal were proportional to a certain constant value, consistent with our theoretical results here.

Qian and Wang [35] investigated the ${\left(\chi N\right)}_{\mathrm{ODT}}$ values for the triblock comb copolymer A*_n_*_+1_(BC)*_n_*. According to their results, the ${\left(\chi N\right)}_{\mathrm{ODT}}$ values for copolymers with the side chain number *n* = 3 was approximately triple the χN for copolymers with *n* = 1 when the volume fractions of the three or two blocks were equal. This phenomenon was also observed in A*_n_*_+1_B*_n_* copolymer [37]. These theoretical calculations are consistent with our results, as ${\left(\chi N\right)}_{\mathrm{ODT}}$ has a linear relationship with architectural periodicity *n* in Equation (2). Owing to the segregation strengths $\chi_{\mathrm{BC}}N=\chi_{\mathrm{AC}}N=0$ used in this part of the calculations, the arm C chains can be deemed as neutral segments embedded in the copolymer sequences. Here the roles of arm C chains are similar to the vacancy lattices in the simulation box in the Monte Carlo (MC) simulations, in which the distribution of vacancy lattices near the interfaces between the incompatible A and B segments plays the role of shielding their repulsion. However, in MC simulations [32,39], the critical ${\left(\chi N\right)}_{\mathrm{ODT}}$ values usually have a reverse relation with the vacancy concentration, as ${\left(\chi N\right)}_{\mathrm{ODT}}\sim {\left(1-f_{C}\right)}^{-1}$, while the value of the fitted exponent becomes −2 for the Janus double-brush copolymers (AC_2_B)*_n_*. Moreover, the ${\left(\chi N\right)}_{\mathrm{ODT}}$ values were found to increase with the volume fraction of the backbone segments [35,37], and our results in Figure 4b are consistent with this.

Palyulin and Potemkin [38,40] investigated the microphase separation of the centipede-shaped copolymers A*_n_*B*_n_*C*_n_*_-1_ with gradient, random, and regular sequences of the branch points. They used a different set of segregation strengths $\chi_{\mathrm{AB}}N=0$ and $\chi_{\mathrm{BC}}N=\chi_{\mathrm{AC}}N=\chi N$. The connectivity of the diblocks into the centipede-shaped copolymers reduces the critical ${\left(\chi N\right)}_{\mathrm{ODT}}$ values at the transition point compared with the linear diblocks. Our calculations may complement theirs, as the segregation strengths are set to be $\chi_{\mathrm{AB}}N=\chi N$ and $\chi_{\mathrm{BC}}N=\chi_{\mathrm{AC}}N=0$ in this part of the calculations. Our calculations indicate that $\chi_{\mathrm{ODT}}$ is influenced by the arm C length $L_{C}$ but nearly not influenced by the architectural periodicity *n*. Thus, $\chi_{\mathrm{ODT}}$ is not changed by the connectivity between the arm C chains of the AC_2_B subunit in the (AC_2_B)*_n_* copolymers. According to Equation (2) and $N=2n\left(L_{A}+L_{C}\right)$, the $\chi_{\mathrm{ODT}}$ value can be expressed as:

(3) $$\chi_{\mathrm{ODT}}=\frac{10.5\left(1+L_{C}/L_{A}\right)}{2L_{A}},$$

Thus, $\chi_{\mathrm{ODT}}$ increases with $L_{C}/L_{A}$, consistent with the results in Figure 4a. Since $\chi_{\mathrm{ODT}}$~1/$T_{\mathrm{ODT}}$, the critical temperature $T_{\mathrm{ODT}}$ for the ODT of such copolymers should decrease with *f*_C_. Calculations from Sanchez et al. [36] indicated that $T_{\mathrm{ODT}}$ was identical for all A*_n_*B*_n_* star copolymers and was not influenced by the architectural periodicity *n* at a volume fraction *f*_A_ around 0.5. Our calculations reached a similar conclusion, i.e., the $\chi_{\mathrm{ODT}}$ or $T_{\mathrm{ODT}}$ values are not influenced by architectural periodicity *n* for the Janus double-brush copolymers (AC_2_B)*_n_* at symmetric compositions *f*_A_ = *f*_B_.

## 4. Conclusions

For the Janus double-brush copolymers (AC_2_B)*_n_* with different architectural periodicities *n* of the repeat subunits AC_2_B, the influences of the topological connection between the AC_2_B star subunits on the phase diagrams and microphase transitions were investigated via a SCFT method. Nine ordered structures were found, including the LAM2, CSH, LAM3, TET2, LAMAB, HEX3, LAMBD, HEX3I, and OOT microphases, at different segregation strengths. Triangle phase diagrams were made and compared between copolymers with different architectural periodicities *n*. The influence of topological connections on the phase diagrams of the copolymers (AC_2_B)*_n_* was found to decrease with *n* and became indiscernible for the copolymers with an architectural periodicity *n* greater than 3. Thus, bond breakage along the copolymer backbone during service had nearly no influence on the phase behaviors of the (AC_2_B)*_n_* copolymers unless their architectural periodicity was below 3. The topological connection between the AC_2_B star subunits improved the critical segregation strengths ${\left(\chi N\right)}_{\mathrm{ODT}}$ at the order–disorder transitions. ${\left(\chi N\right)}_{\mathrm{ODT}}$ was found to have a linear relationship with architectural periodicity and ${\left(1-f_{C}\right)}^{-2}$ for the (AC_2_B)*_n_* copolymers with the neutral backbone. Moreover, $\chi_{\mathrm{ODT}}$ increased linearly with the length ratio between the arm C and arm A chains for the Janus double-brush (AC_2_B)*_n_* copolymers.

The future research plan is to investigate the influence of periodicity in molecular architectures on the interfacial properties of the Janus double-brush copolymers.

## Figures and Tables

**Table 1:** The ordered morphologies, abbreviated names, and their symbols used in the triangle phase diagrams for the microphases of the Janus double-brush copolymers (AC_2_B)*_n_*. Using a four-arm AC_2_B star subunit as an example, the chain packing conformations in these morphologies are presented in the last column. Besides these symbols, we use 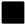 to represent the “disordered” phase (DIS) and use 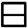 to represent the “two-color lamellar” phase (LAM2).

Snapshots	Abbreviated Names	Symbols	Conformations
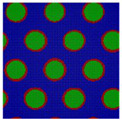	CSH	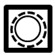	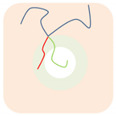
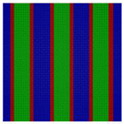	LAM3	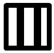	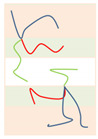
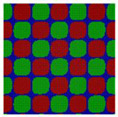	TET2	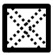	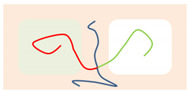
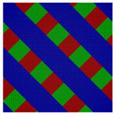	LAMAB	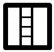	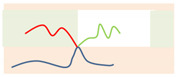
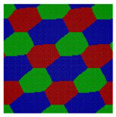	HEX3	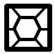	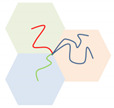
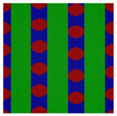	LAMBD	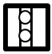	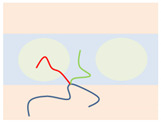
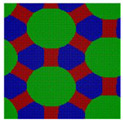	HEX3I	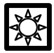	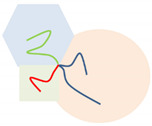
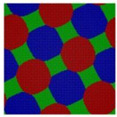	OOT	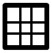	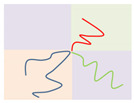

## Data Availability

The data presented in this study are available on request from the corresponding author.

## Appendix A

The abbreviations and variables used in this paper are listed in Table A1 and Table A2, respectively, as follows:

**Table A1:** The abbreviations used in this paper and their full names.

Abbreviation	Full Name
2D	2 Dimensions
3D	3 Dimensions
BDO	1,4-Butanediol
[Bmim][PF_6_]	Hydrophobic ionic liquid 1-butyl-3-methylimidazole phosphorus hexafluoride
CSH	Core-shell hexagonal lattice phase
DIS	Disordered phase
FFTW	Fast Fourier Transform in the West
GPC	Gel permeation chromatography
HEX3	Three-color hexagonal honeycomb phase
HEX3I	Hexagon outside “two-color” hexagonal lattice phase
LAM2	“Two-color lamellar” phase
LAM3	Three-color lamellar phase
LAMAB	Lamellar phase with alternating beads
LAMBD	Lamellar phase with beads inside
MC	Monte Carlo
MDI	Diphenyl methane diisocyanate
ODT	Order–disorder transitions
OOT	Octagon-octagon-tetragon phase
UV	Ultraviolet
PDMA	Poly(*N,N*-dimethyl acrylamide)
PDMA-*b*-PMMA	Diblock copolymer containing the poly(*N,N*-dimethyl acrylamide) and poly(methyl methacrylate) blocks
PMMA	Poly(methyl methacrylate)
PU	Polyurethane
PU-*g*-PDMA/PMMA	Copolymer consisting of the PDMA and PMMA chains grafted at the same reactive site along the PU backbone
PU-*g*-PDMA_48_/PMMA_50_	The degree of polymerization for the PDMA and PMMA chains is 48 and 50, respectively, in the copolymer
SCFT	Self-consistent mean field theory
TEM	Transmission electron microscope
TET2	Two interpenetrating tetragonal lattice phase

**Table A2:** The quantities and all variables used in this paper and their physical meanings.

Variable	Physical Meaning
*η*	Lagrange multiplier to ensure an incompressibility constraint
*F*	The free-energy density
*f_i_*	Composition of the *i* segments in the copolymer, *i* = A, B, C...
*k*_B_	Boltzmann constant
$k_{B}T$	Thermal energy
χ	Flory–Huggins dimensionless exchange energy
$\chi_{ij}$	Flory–Huggins interaction parameter between dissimilar segments *i* and *j*, where *i*, *j* = A, B, C...
$\chi_{\mathrm{ODT}}$	Flory–Huggins dimensionless exchange energy at the order–disorder transition
χN	Segregation strength
$\chi_{ij}N$	Segregation strength between dissimilar segments (blocks) *i* and *j*, where *i*, *j* = A, B, C...
χN/n	The averaged segregation strength for one repeat subunit in the copolymer
${\left(\chi N\right)}_{\mathrm{ODT}}$	Critical segregation strength for the order–disorder transitions
*L_i_*	Length of the arm *i* chain, *i* = A, B, C...
$L_{C}/L_{A}$	Ratio between the lengths of the arm C and arm A chains
$L_{\max}$	Critical ratio between the lengths of the arm C and arm A chains
*m*	Total number of chains in the system
*n*	Architectural periodicity
*N*	Total chain length of the copolymer
$N_{0}$	Length of one repeat subunit in copolymer
$\phi_{i}\left(r\right)$	The local volume fractions at position r for the *i* segments, *i* = A, B, C...
Q	The partition function
q	The end-segment distribution function
$q^{+}$	The conjugate of the end-segment distribution function
r	The spatial coordinate vector
$R_{g}$	The copolymer’s radius of gyration
σ	Segment length
T	Temperature of the system
$T_{\mathrm{ODT}}$	Critical temperature for the order–disorder transition
V	The volume of the system
$\omega_{i}$	Hypothetical external potentials for the *i* segments, *i* = A, B, C...
